# The growing effect of job demands on teacher mental health: results from a longitudinal national household panel survey

**DOI:** 10.1186/s12889-025-22372-5

**Published:** 2025-05-16

**Authors:** Richard W. Morris, Lisa E. Kim, Alyssa Milton, Nick Glozier

**Affiliations:** 1 ARC Centre of Excellence for Children and Families over the Life Course, Sydney, NSW Australia; 2 Central Clinical School, Faculty of Medicine and Health, University of Sydney, Sydney, NSW Australia; 3 School of Psychology, University of Sydney, Sydney, NSW Australia

**Keywords:** Mental health, Workplace, Risk factors, Job demands, Job control, Occupational mental health

## Abstract

**Background:**

Teacher mental health is an important predictor of student outcomes and teacher workforce retention, and has been declining for some years, exacerbated by the COVID-19 pandemic. The various causes of this trend have been speculated to include a workforce that is younger and less experienced, as well as increasing work demands.

**Methods:**

We evaluated the trends in teacher mental health between 2005 to 2022, using the 5-item Mental Health Inventory (MHI-5) from the annual Household Income and Labour Dynamics in Australia (HILDA) survey. We tested whether the trend was due to changes in non-work related factors (i.e., changes in workforce composition), or due to workplace risk factors (i.e., high job demands and low autonomy).

**Results:**

Teacher mental health was stable to 2011 then declined from a median of 80 (IQR 68–88) to 76 (IQR 60–97) MHI-5. The decline was not explained by changes in the workforce composition. The prevalence of high job demands was stable over this period (53% to 55%) while low autonomy and control increased from 34 to 58%, especially after 2018. At the same time, the strength of the association of high job demands with poor mental health increased from 1.32 [95%CI -0.45 to 3.09] MHI-5 units to 4.91 [3.34 to 6.47] MHI-5 units.

**Conclusions:**

The decline in teacher’s mental health was partly explained by an increasing sensitivity to job demands. Given the reported level of demands did not increase, addressing the reduction in job autonomy over time (which enables workers to cope with high demands) may improve policies to support teacher mental health and workforce retention.

**Supplementary Information:**

The online version contains supplementary material available at 10.1186/s12889-025-22372-5.

## Background

The mental health and wellbeing of teachers is an important predictor of student outcomes, workforce retention, as well as a broader indicator of our socioeconomic priorities [1–5]. Recent changes and challenges to public schooling around the world, including the global COVID-19 pandemic (e.g., [6]), have highlighted the connection between job stressors and mental health in teachers (as well as in other public services such as hospitals and nurses) [7].

The teaching workforce in Australia, like in many other countries, is predominantly female (72%) [8], with an average salary less than that of other degree-qualified, non-managerial adult employees [9]. Over the decades, various educational reforms have impacted teachers’ professional lives. For example, there has been a gradual shift towards increased school autonomy, which has increased teachers’ personal accountability and administrative workload and affected the nature of relationships within schools [10, 11]. Additionally, the school inspection model and class size standards have changed with the increasing power devolution, where each state and territory have its own model of inspection and norms. At the same time, despite recent pay increases, the teacher pay scale in Australia is relatively flat such that older teachers earn up to as much as $AUD50K less than other bachelor-degree employees [9].

Teachers’ wellbeing has become a growing concern among practitioners and policymakers, highlighted by troubling statistics. For instance, the Teaching and Learning International Survey (TALIS) revealed that only 45 percent of lower secondary teachers in Australia reported feeling valued by society [12, 13]. Moreover, nearly 60 percent of Australian teachers reported experiencing significant levels of stress, a figure higher than the OECD average of nearly 50 percent, with high administrative load been identified as a major source of stress [12, 13].

The global COVID-19 pandemic and its implications have also been a source of poor wellbeing among teachers. For example, a meta-analysis of 54 studies involving 256,896 teachers across 22 countries found high prevalence of stress (62.6%), anxiety (36.3%) and depression (59.9%) during 2020/2021 [14, 15]. However, longitudinal studies of teacher mental health have revealed declines started earlier than the COVID-19 outbreak in many countries. A longitudinal national household panel survey in Australia found that teachers’ mental health may have begun to decline as early as 2015, and this decline was exacerbated during the COVID-19 pandemic in 2020 [16]. The problem was not unique to Australia and has been found in other countries. For example, a study of multiple household panel surveys in the UK found teachers have been reporting declines in mental health since 2013, along with concomitant increases in mental illness and antidepressant use [17]. Since then, a qualitative trajectory analysis of teachers in the UK found consistent declines in their mental health during 2020 [18], suggesting the COVID-19 pandemic likely exacerbated the ongoing trends in the UK.

Thus both local and global reasons for the decline in teacher mental health have been proposed, including work demands as teachers face increasing pressure to meet administrative load and diverse student needs [10, 18, 19]; a younger lesser experienced workforce, who face greater challenges in managing classroom demands [19, 20] and are a demographic more vulnerable to mental health issues [21, 22]; and systemic issues such as lack of management and administrative support [18, 23]. However, widespread declines in the mental health of the general adult population in the last ten years have made it difficult to attribute declines among any specific occupational group to work-related factors. For instance, Hoang [16] found teacher mental health did not decline significantly more than general employees in the wider population over the same period. Likewise, Jerrim [17] reported widespread increases in mental health diagnoses and antidepressant use across occupational groups in the UK, consistent with a general increasing willingness to diagnose and disclose mental health problems in the wider population.

Regardless of how unique any such declines are to teachers, the causes of such declines may differ among occupations. Changes over time in teacher mental health could be due to (a) changes in composition of the workforce, (b) changes in the nature of teaching, or (c) changes in the vulnerability of teachers. Changes to the workforce composition — such as an increasing proportion of young people or women who are more likely to report lower mental health [21] — could produce an observed decline in the average level of teacher mental health (without any corresponding changes in individual levels of mental health). Changes in the nature of teaching, such as working conditions, might also explain the decline in teacher mental health. High stress environments, high workloads and low employee autonomy, have been identified as risk factors for poor physical and mental health [24–27]. For teachers, specific risk factors include long working hours, high administrative load and lack of teaching experience [28, 29]. Exposure to workplace risk factors is commonly indicated by reports of high job demands and low job control (i.e., low autonomy) on employee questionnaires [e.g., [30]]. Workers (including teachers) suffering both high job demands and low job control (i.e., “job strain”) are at greater risk of anxiety and depression, burnout, reduced job satisfaction, and cardiovascular disease [31, 32].

Exposure to workplace risk factors could explain the decline in teacher mental health in at least two ways. First, the prevalence of workplace risk factors may be increasing (due to job changes, workplace changes, or changes in working conditions), and this would represent changes in the nature of teaching. Alternatively, or in addition, the decline in teacher mental health may be due to increased vulnerability to the risk factors already present. For instance, it has been suggested that young teachers have become less resilient to the demands of the workplace since COVID-19 due to the lack of training, remote teaching, and support from coworkers [33, 34]. A decline in tecaher workplace autonomy (i.e., job control) could also expose them to the ill effects of high job demands that were already prevalent but otherwise unchanged [27]. Thus increased vulnerability of teachers may manifest in lower average levels of teacher mental health, even if the workplace had not changed and prevalence of workplace risk factors remained constant.

Therefore, the aim of the present study was to identify whether the teaching job has changed or whether teachers themselves have changed. We determined whether the trends in the mental health of teachers was due to (a) broader demographic changes in the sex or age composition of the teaching workforce; (b) changes in the reported exposure to work risk factors (i.e., the teaching job has gotten worse); and (c) whether teachers have become more vulnerable or less resilient to such stressors over time.

## Methods

Explanatory factors for the long-term trends in the mental health of school teachers were evaluated using regression models. Additive models with (penalised) smooth terms for time allowed us to capture complex temporal trends in mental health. To identify which factors were responsible for declines in teacher mental health, terms for non-work related factors (such as age and sex) and work-related factors (such as job control and job demands) were added consecutively, and the resulting trends were then evaluated for changes in trajectory. Furthermore, we compare the trends observed for teachers with another occupational group—nurses and midwives (hereon *nurses*), to help identify occupation-specific effects. Nurses are a relevant comparison group to teachers as they are also designated as essential public service employees, with similar educational requirements and renumeration (and similar demographic composition)—who share comparable levels of average mental health [16]. Nurses also show similar trends as teachers in workers compensation claim rates [35], a key indicator of job stress and thus making them a particularly relevant comparator.

### Sample

The HILDA survey, funded by the Australian government, was collected yearly between 2001 to 2022. The sampling frame is a nationally representative probabilistic sample of Australian households which collects information on a wide range of aspects of life in Australia, including health and wellbeing. The longitudinal design included 7,682 households in Wave 1 (66% response rate), with 87% retention in Wave 2 and over 90% retention at subsequent waves. The survey adds children (15-years and above) born to sample members and people who joined or shared a household with a sample member. In Wave 11, an additional 2,153 households were included to correct for population growth and alleviate bias from non-random attrition (*n* = 4,009) [36]. The focus of this paper is from Wave 5 (2005) to Wave 22 (2022), which contain all 17 items measuring workplace psychosocial stressors (see below).

### Occupational definitions by ANZSCO professional codes

Occupation was ascertained by the current occupation response and coded to the Australian and New Zealand Standard Classification of Occupations 4-digit codes (Australian Bureau of Statistics, 2009 ANZSCO First Edition, Revision 1). The two codes for “Primary” and “Secondary” school teachers were used. The 4-digit code for “Nurses & Midwives” was used for comparison, and includes hospital and non-hospital nurses.

### Outcome—Mental health

The MHI-5 is a well-validated instrument for assessing mental health in population research [37–39]. In Australia it has been used as a proxy for common mental disorders where it has high levels of sensitivity and specificity, particularly for mood disorders [40], and trends in the full distribution of scores correspond with the prevalence of mental illness in HILDA when using a prescribed cut-off value (see Figure A1 in Supplementary). The MHI-5 is collected every year in the HILDA survey, and consists of five items assessing positive and negative aspects of mental health. Respondents are asked to state how often they have experienced each of the following during the past four weeks. The items were: 1) “Been a nervous person”, 2) “Felt so down in the dumps nothing could cheer you up”, 3) “Felt calm and peaceful”, 4) “Felt down”, 5) “Been a happy person”.

The response to each item is selected from a 6-point scale “All of the time”, “Most of the time”, “A good bit of the time”, “Some of the time”, “A little of the time”, “None of the time”. Each response is scored 0 to 5, and items were recoded so that higher scores indicated better mental health. Raw scores were summed across the items and then linearly transformed to a 0–100 scale [41]. In accordance with the manual a person-specific score was estimated in any year on which there were valid responses on three or more items, the average being calculated and applied to missing items [42].

### Workplace psychosocial stressors

Psychosocial stressors, which at extreme levels are major risk factors for poor mental health [30], were derived from a set of 17 questions representing *job demands & complexity* (hereon “job demands”) and *job control* [43], which were collected annually from 2005. The subset of items contributing to each component are shown in Supplemental Tables A1 to A2. We constructed a component score for each person in each year by calculating the (within-person) average of the component items (after reverse scoring the negatively worded items). Missing responses were implicitly imputed by this mean estimation procedure.

### Analysis

The prevalence of each risk factor was determined by the proportion of people reporting high job demand, or low job control in each year. Fixed thresholds for high and low component scores were defined by the most extreme quartile of responses in the entire employed sample of HILDA (all occupations among all employed people in all eighteen waves), so as to provide a common threshold value across the two professions examined here. Thus the fixed threshold for low job control was a job control score < 3.25, and the fixed threshold for high job demand was a job demand score > 4.55. Significant linear changes over time in the prevalence of each risk factor were tested in a weighted linear regression (i.e., equivalent to an aggregated linear probability model).

Trends in the mental health of teachers and nurses between 2005 to 2022 were modelled as a smooth function of time for each occupation. The smooth trend for each occupation was estimated by a penalized, non-linear spline function over time (years) in an additive model [44, 45]. Both linear and non-linear effects of time were included in Model I, and the resulting smooth trend represented the unadjusted mental health trend over time for each occupation.

We then modelled trends adjusted for the potential confounding effect of demographic shifts. Model II estimated the smooth trend of mental health, after including linear terms for age, sex and job tenure and their interaction with time. Thus this model adjusted for changes in non-work related factors over time by holding each constant at their mean value. Job tenure was determined from the HILDA variable derived from each persons time with their current employer cross-referenced against any occupation changes since the last interview.

Model III added the effect of job control in addition to the terms described for Model II. Model IV added the effect of job demands in addition to the Model II terms. In both models, the levels of job demands and job control were added as parametric interaction terms with time, to account for non-linear changes. From Models II to IV we produced a smooth trend for mental health, which represented the conditional mental health trend after holding the other terms constant. We present the smooth mental health trends as zero-centered partial effects to allow comparison and identify trajectory differences due to the effect of each additional term (i.e., workplace psychosocial stressor). See Supplemental B for all model definitions.

### Temporal changes in the sensitivity of teacher mental health to job demands and job control

The sensitivity of teachers’ mental health to workplace psychosocial stressors was determined by estimating and comparing the absolute slopes ($\left|\beta \right|$) of each job component score (job demands and job control), from a model predicting mental health (MHI-5 scores). MHI-5 scores were modelled using an additive model [44, 45], with a (penalized) parametric interaction term for each job component score and time. We included the effects of both job demands and job control in a single model and present the slope of each job component when the other job component is held constant. This allows us to uniquely identify the changing impact of each workplace psychosocial stressor on teacher mental health over time. The model also estimated the uncertainty around each slope, which results in confidence intervals with close to nominal (frequentist) coverage properties [44]. The greater each slope deviates from zero over time ($y=0$), the greater the sensitivity of mental health (in MHI-5 units) to that particular workplace psychosocial stressor.

Along with comparing the slopes for each job component, we also determined how much variance (${R}^{2}$) in mental health was explained by each job component in each year by ordinary least square (OLS). This tells us whether the overall importance of workplace psychosocial stressors for mental health has changed over time.

## Results

The number of teachers identified by ANZSCO code in each year ranged from *n* = 332 (2008) to *n* = 434 (2011). Nurses ranged from *n* = 180 (2005) to *n* = 274 (2020). Table 1 shows the majority of teachers were female, representative of the Australian teacher population [71.9%, 9], and were similarly distributed over primary and secondary education sectors [50.6%/49.4%, 9], while the geographic distribution favored city schools over regional and remote schools. Teachers and nurses had significant increases in education levels, and income (adjusted for 2022 dollars) between 2005 and 2022. Gender, partnership and job tenure showed no differences in either occupation sample over the 18 years. Both occupations remained female dominated, and experienced similar levels as well as significant declines in mental health between 2005 and 2022. The prevalence of low job control increased between 2005 and 2022 among both teachers and nurses. The prevalence of high job demands among teachers was consistently high over the period (greater than 50%), whilst an increasing proportion of nurses reported high demands.

**Table 1:** Change in demographic composition between 2005 and 2022

Group	Characteristic	2005^a^	2022^a^	*p*-value^2^
Teachers	Total	*N* = 349	*N* = 413	
	Female	253 (72%)	293 (71%)	0.6
	Age	44 (35, 51)	41 (32, 52)	0.2
	Coupled	260 (74%)	319 (77%)	0.4
	New parent	50 (14%)	79 (19%)	0.078
	Edu			< 0.001
	Postgraduate	27 (7.7%)	87 (21%)	
	Graduate diploma	119 (34%)	94 (23%)	
	Bachelors degree	126 (36%)	178 (43%)	
	Year 12	75 (21%)	54 (13%)	
	Year 11 or below	2 (0.6%)	0 (0%)	
	Tenure (years)	9 (3, 18)	8 (3, 17)	0.3
	Sector			0.8
	Primary	191 (55%)	229 (55%)	
	Secondary	158 (45%)	184 (45%)	
	Region			0.10
	City	225 (64%)	279 (68%)	
	Regional	91 (26%)	83 (20%)	
	Remote	33 (9.5%)	51 (12%)	
	Real household income ($000 s)	61 (47, 73)	76 (60, 97)	< 0.001
	Mental health	80 (68, 88)	76 (64, 84)	< 0.001
	Low job control	111 (34%)	218 (58%)	< 0.001
	High job demand	161 (50%)	200 (53%)	0.4
Nurses	Total	*N* = 180	*N* = 270	
	Female	165 (92%)	244 (90%)	0.6
	Age	42 (35, 48)	38 (29, 53)	0.2
	Coupled	132 (73%)	192 (71%)	0.6
	New parent	35 (19%)	43 (16%)	0.3
	Edu			< 0.001
	Postgraduate	4 (2.2%)	32 (12%)	
	Graduate diploma	42 (23%)	63 (23%)	
	Bachelors degree	84 (47%)	139 (51%)	
	Year 12	38 (21%)	36 (13%)	
	Year 11 or below	12 (6.7%)	0 (0%)	
	Tenure (years)	5 (2, 13)	6 (2, 13)	0.3
	Region			0.8
	City	115 (64%)	180 (67%)	
	Regional	42 (23%)	60 (22%)	
	Remote	23 (13%)	30 (11%)	
	Real household income ($000 s)	58 (47, 72)	79 (61, 96)	< 0.001
	Mental health	80 (68, 84)	76 (64, 84)	0.012
	Low job control	46 (27%)	99 (39%)	0.010
	High job demand	56 (33%)	139 (55%)	< 0.001

^a^N = N; n (%); Median (IQR)

^2^Pearson's Chi-squared test; Wilcoxon rank sum test; Fisher's Exact Test for Count Data with simulated *p*-value (based on 2000 replicates)

### Survey results

#### Yearly mental health levels and prevalence of workplace risk factors

**Fig. 1:**
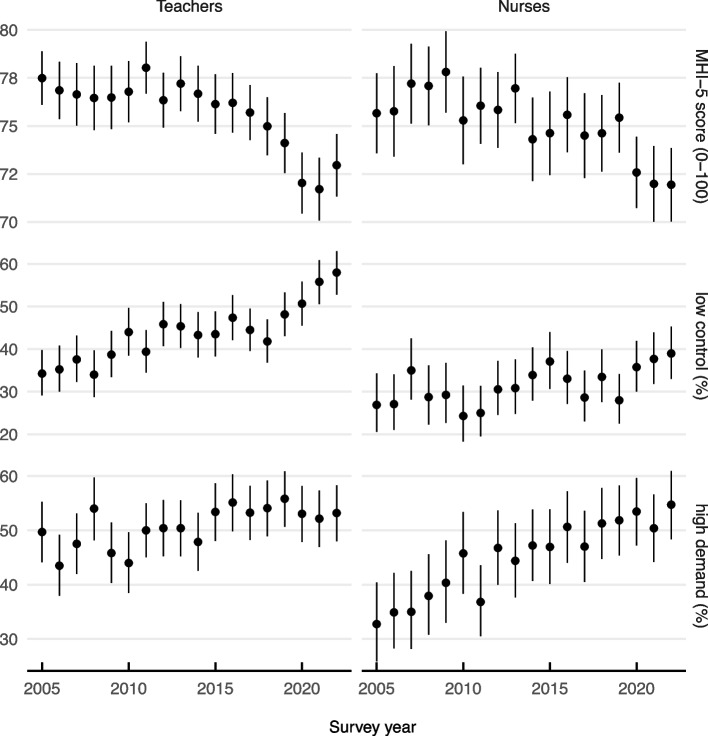
Annual population prevalence (%) of psychosocial risk factors and mental health levels (MHI-5 scores). Mean MHI-5 scores (0–100) and prevalence (%) of people reporting low job control and high job demand in each year (where high and low are defined by the job component score thresholds between the most extreme quartile of the employed population). Vertical lines represent 95 percent confidence intervals

Figure 1 presents the mean mental health levels (MHI-5 scores) in each year, along with the yearly prevalence of low job control and high job demands, for each profession. Mental health levels decreased sharply among teachers after 2011, with year-on-year decreases between 2013 to 2021 (see trend analysis below). At the same time, the prevalence of low job control increased among teachers, at an average rate of 1% per year ($\beta =1.13,p<.001$), while prevalence of high job demands increased at less than half a percent ($\beta =0.47,p=.002$) and so was relatively stable. Among nurses, the increases in prevalence of low job control and high job demands were less than 1% per year ($\beta =0.53,p=.005$ and $\beta =0.97,p<.001$, respectively).

#### Mental health trend differences due to workplace psychosocial stressors

**Fig. 2:**
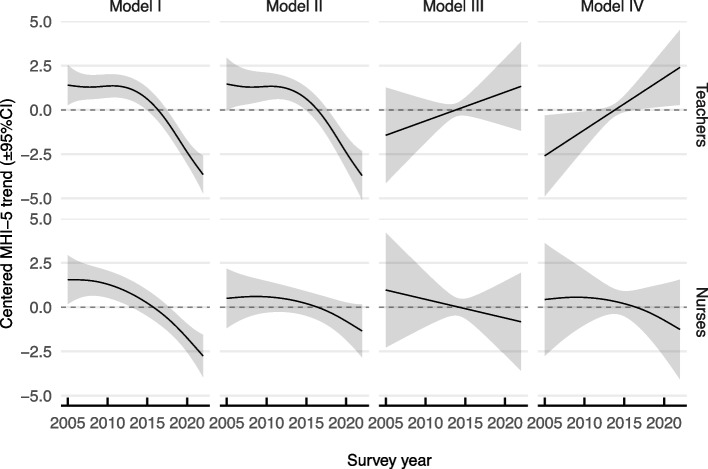
Mental health trend differences due to workplace psychosocial stressors. Unadjusted and adjusted effects of year (± 95%CI) on mental health from each model. Model I is the unadjusted trend over time (i.e., the mental health trend). Model II is the trend adjusted for non-work related factors (age, sex, length of job tenure) held constant at their mean value. Model III is adjusted for the same non-work related factors as well as job control held constant at the highest value. Model IV is adjusted for job demands held constant at the lowest value (and the non-work related factors). Shaded areas represent 95 percent confidence intervals

We estimated the total smooth trend in mental health for teachers and nurses (Model I), as well as an adjusted trend after taking into account changes in the composition of age, gender ratio, and job tenure over the period as non-work related controls (Model II). These non-work related controls were included because we know that mental health scores of the general population change with age and gender in this data [21], and job tenure is also likely associated with mental health. In addition to these controls, Models III and IV adjusted for the levels of workplace psychosocial stressors (job control and job demands, respectively). Smooth trends and 95% confidence intervals are shown in Fig. 2.

Model I shows the mental health (MHI-5 scores) of teachers and nurses declined over the period, with a significant non-linear change after 2011 (${F}_{3.68}=22.99,p<.001$ and ${F}_{2.62}=12.88,p<.001$, respectively). Model II shows that once non-work related factors were held constant then most of the estimated decline among nurses was explained (${F}_{1}=1.76,p=.17$); while the estimated decline among teachers was still apparent (${F}_{3.75}=10.79,p<.001$). A supplementary analysis provided no evidence of state-specific effects in mental health trends (AIC & BIC in favor of a non-specific model were 1.9 and 98.2, respectively, see Supplemental Figure D1 and Table D1). Models III and IV show the estimated mental health decline among teachers was eliminated by adjusting for job control (${F}_{1.0}=1.49,p=.28$), while the estimated decline was not just eliminated but reversed when job demands were removed (${F}_{1.69}=5.67,p=.01$). The trends in nurse mental health remained non-significant ($p=0.54$ and $0.41$, respectively), and a follow-up linear mixed model with random intercepts and effects of time for each participant confirmed the (fixed) linear effect of time was also non-significant for the ${\beta }_{time}$ of nurses (${t}_{479.8}=-0.86,p=.39$). This suggests that the decline in teacher mental health since 2011 can be explained by changes in workplace psychosocial stressors, while the trends in nurse mental health are most likely due to demographic changes to the workforce over time.

### Temporal changes in the sensitivity of teacher mental health to job demands and job control

**Fig. 3:**
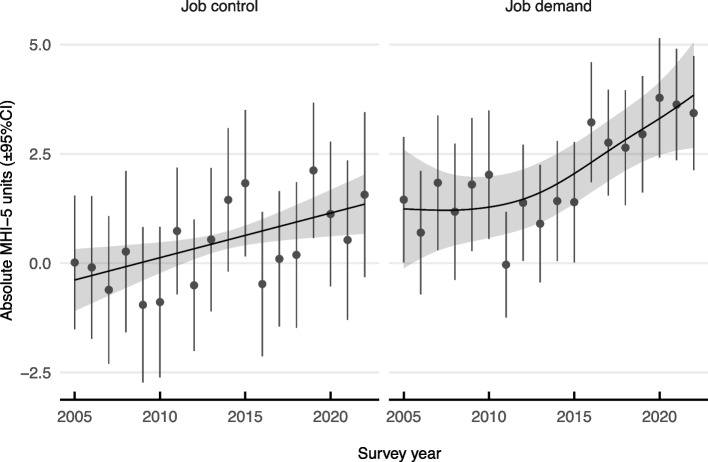
Sensitivity of teacher mental health to job demands and job control. Penalized (smooth shaded region) and unpenalized (points and vertical lines) slopes (± 95%CI) of each job component on teacher mental health, representing sensitivity (change in absolute MHI-5 units) of mental health to psychosocial stressors between 2005 and 2022. Where each 95% confidence interval excludes zero (shaded or error-bar) indicates where teachers became more sensitive to that component over time or in that year

We estimated the association of teacher mental health with both job components in a regression model of mental health by year, where the absolute slope of each job component in each year ($\left|\beta\right|$) represents the sensitivity of mental health to workplace psychosocial stress. Figure 3 presents the change in sensitivity over time among teachers. The smooth trend represents the penalised (partial) effect of each psychosocial stressor on mental health over time, and the solid points represent the unpenalised point estimate (OLS) in each year. Sensitivity is indicated by the deviation from  $y=0$ in each trend or point in absolute MHI-5 units.

Figure 3 shows the increasing impact of a 1-unit change in each workplace psychosocial stressor over time (holding the other stressor constant). *Ceteris parabis*, the trends show the sensitivity of teacher mental health to job demands increased more than job control. The inflexion of the increase in sensitivity to job demands after 2011 is consistent with the observed decline in teacher mental health at the same point.

The fit (${R}^{2}$) of the OLS regression of job components on mental health each year improved from ~ 4% in 2005 to almost ~ 16% by 2022 (see Figure B1 in Supplementary). The improvement in the ${R}^{2}$ suggests that job control and job demands explain an increasing proportion of the variation in the mental health of teachers in the past eighteen years, however the importance of job demands increased faster than job control.

In combination with the stable rate of prevalence of high job demands over time (Fig. 1), this result represents evidence that the decline in teacher mental health is best explained by changes in sensitivity to the stress of job demands among teachers rather than due to changes in the prevalence of high job demands. Supplementary analyses of teachers’ happiness, real wages, or job skills also did not reveal any evidence the decline in mental health was explained by changes to the effort-reward ratio [46] or job resources [47, 48], as predicted by recent updates to the Job Demand-Control model (Supplemental C). Given the loss of teacher autonomy (Fig. 1, low job control prevalence) and job control’s important role as a buffer against high demands (Fig. 2, Model III), the most likely reason for the increased sensitivity to job demands is the loss of autonomy among teachers in the workplace.

## Discussion

The mental health of teachers has markedly declined since 2011, while the prevalence of workplace risk factors has accumulated over years and underscores the growing pressure on the profession. In particular, high job demands were constantly prevalent (> 50%) among teachers between 2005 and 2022, while prevalence of low job control increased markedly (34% to 58%) (Fig. 1). However the observed changes in any one risk factor alone are not sufficient to explain the sharp decline in teacher mental health after 2011. Instead, the current findings indicate that the decline in teacher mental health is best explained by increased sensitivity to high job demands due to the loss of job control. By contrast, the mental health decline of nurses was explained by changes in the workforce composition (Fig. 2). These findings are important as employee mental health, which can be considered as a type of *job resource* [47], predicts motivation to leave the profession and employee turnover [5].

One of the most striking findings in the current study is the increasing sensitivity of teachers’ mental health to the demands of the teaching job (Fig. 3). Despite over half the teachers in the HILDA survey reporting high job demands each year, the impact on mental health only appeared after 2011. That is, the same level of job demands that was manageable before 2011, contributed more substantially to mental health decline among teachers after 2011 and in particular after 2015 (Fig. 3, right panel). Typically the level of job control or autonomy acts as an important buffer against the ill effects of high job demands [27], and we found the increased prevalence of low job control also increased after 2015 (Fig. 1). Yet job control by itself (i.e., in the absence of high job demands) will not produce changes in job strain or mental health [27]. Consistent with this, our analysis of mental health trend differences demonstrated that holding job control constant (at the highest level) prevented the decline in teacher mental health but did not reverse it. Moreover, our sensitivity analysis showed that job control did not increase in importance over job demands (Fig. 3, left panel), consistent with its inhibitory role on the ill effects of job demands but not acting directly on mental health. The implication is that the job demands of teaching, which have always been high, have become less controllable. Thus efforts to improve teaching job control may be an important lever to slowing the decline in teacher mental health.

We found that adjusting for job demands alone was sufficient to reverse the decline in teacher mental health, supporting the consensus view that interventions aimed at reducing teacher job demands would be particularly effective in improving mental health outcomes. Common job demands in the teaching profession include a high workload and having multiple roles beyond teaching, such as administrative duties and responsibility for student physical and mental wellbeing [18, 23]. Given that teachers in Australia reported greater amount of time spent on planning, marking and administrative tasks and also reported higher levels of stress, policymakers have focused on methods to reduce the number of hours teachers need to spend on non-teaching tasks [13]. One of the priorities of the Australian government in the National Teacher Workforce Action Plan [49] is to reduce teacher workload through the Workload Reduction Fund and to evaluate the effectiveness various methods used to reduce workload. Similar endeavors are being pursued in other countries such as the UK Government’s Department for Education, which has published guidance and resources to assist schools in reducing workload to improve teacher wellbeing (Department for Education, 2024a). Recommendations to reduce teacher workload included the removal of tasks and activities that do not require teachers’ professional skills and judgment (Department for Education, 2024c).

The present results also suggest that alternative strategies to improve teacher resilience to the high job demands could focus on improving personal autonomy and the decision-latitude of teachers. Strategies include offering more control over teaching methods, classroom management and curriculum design, as well as flexible scheduling, job sharing and part-time positions to help self-manage workload. By providing choice over when and how teachers complete their duties, schools can help reduce the negative impact of high job demands when they can’t be reduced. In addition, improving contextual resources, such as management or coworker support, or creating a trusting and positive school climate, can also help increase teacher’s resilience [48, 50]. Unfortunately HILDA does not survey coworker support and so any role in the results reported here must remain subject to future research using other data. Likewise, increasing teachers pay may act as an important buffer to job stress, as predicted by the Effort-Reward Imbalance model [46]. We do not report the role of teacher pay (but see Supplemental C), which has been declining relative to other professions since the 1980s, however this may be an important mediator of the present effects [9]. In general, contextual resources such as coworker support and relative pay are also important buffers of job demands beyond job control, and may govern whether interventions are successful.

### Limitations

We have focused on individual level factors, however a systematic review on teacher resilience interventions recommended that increasing teacher resilience should be tackled at multiple levels [50]; i.e., person, microsystem, mesosystem, and exosystem, as an individual is a function of multiple levels [51]. Regional differences and state-specific effects may exist in the present results, because of increasing school autonomy in Australia and the state and territory governments are responsible for running schools and setting local policy. HILDA is collected from every state and territory in Australia and a supplementary analysis to detect state-specific trends in teacher mental health did not indicate significant differences (Supplemental D). Likewise there were no significant differences detected in mental health trends among primary and secondary school teachers, nor between teachers in city, regional or remote schools. However caution should be taken when generalizing the study findings to other countries which may experience different contextual factors or the socioeconomic context is different. Other limitations include our focus on self-reported measures of mental health symptoms rather than help-seeking or treatment, which was a deliberate design decision of this study because symptoms are not confounded with availability of medical care (but they do not necessarily correspond with clinical diagnoses or represent the economic cost of care). Finally, despite the longitudinal analysis, our methods are ultimately based on observational data and so the results are correlational and the conclusions are descriptive. As such, while policy development must (partially) depend on observational studies, interventions should be developed with caution. For instance, the teacher mental health decline could represent the loss of support or buffers in other (unsurveyed) areas of the workplace (such as coworker support).

## Conclusion

Overall our findings indicate that while the nature of teaching has remained relatively stable, teachers have become more vulnerable to its demands. The decline in teacher mental health has significant implications beyond the individuals directly affected, including students and the wider educational system. Beyond reducing job demands, practioners and policymakers should consider how individual teacher autonomy (among the wider workplace resources) can be provided to improve resilience to the high job demands and help teachers thrive personally and professionally.

## Supplementary Information

Supplementary Material 1.

## Data Availability

All code used in the production of this manuscript is available at https://github.com/datarichard/modern-teachers. The HILDA survey data is available by request from the DSS Longitudinal Studies Dataverse (https://dataverse.ada.edu.au/dataverse/DSSLongitudinalStudies). DOI: 10.26193/YP7MNU.

## Abbreviations

HILDA: Household Income and Labour Dynamics of Australia
MHI-5: 5-Item Mental Health Inventory
ANZSCO: Australia and New Zealand Standard Classification of Occupations
OLS: Ordinary Least Squares
UK: United Kingdom
DSS: Department of Social Services
