# Supplementary material for: The growing effect of job demands on teacher mental health: results from a longitudinal national household panel survey
Source: BMC Public Health. 2025 May 16;25:1810. doi: 10.1186/s12889-025-22372-5 (PMC12083033; doi:10.1186/s12889-025-22372-5)
Supplement: Supplementary file 1 — Supplementary Material 1. [file 12889_2025_22372_MOESM1_ESM.pdf]

The growing effect of job demands on teacher mental health: results from a longitudinal national household panel survey

SUPPLEMENTAL MATERIAL

Feb 21, 2025

Supplemental A. Variable definitions

Many surveys of occupational wellbeing, or items focused on work-related wellbeing tend to produce biased estimates due to self-selection of employees unhappy with their job and seeking to express their concerns or grievances in a single-issue survey response. Even large supposedly 'representative' samples can overrepresent unhappy respondents by more than 2.5 times due to such self-selection incentives into work-related surveys [1,2]. We used HILDA which does not suffer this problem due its wide topic coverage and probabilistic sampling.

HILDA also incorporates a comprehensive set of questions concerning mental health including the MHI-5, the focus of this study, as well as the K10 which measures psychological distress [3]. Both instruments have been validated for assessing mental health in population research [4–6]. Both have high levels of sensitivity and specificity for detecting mood disorders in the community, where cut-offs have been prescribed and validated against clinical diagnostic criteria [7]. Figure A1 presents the trends in MHI-5 score against the prevalence of mood disorders according to the cut-offs provided by [7]. For the purpose of comparison, the MHI-5 score was reversed to represent mental *ill-health*.

Figure A1. Trends in mental ill-health (reversed MHI-5) and prevalence of mood disorders (MHI-5) in HILDA 2005-2022

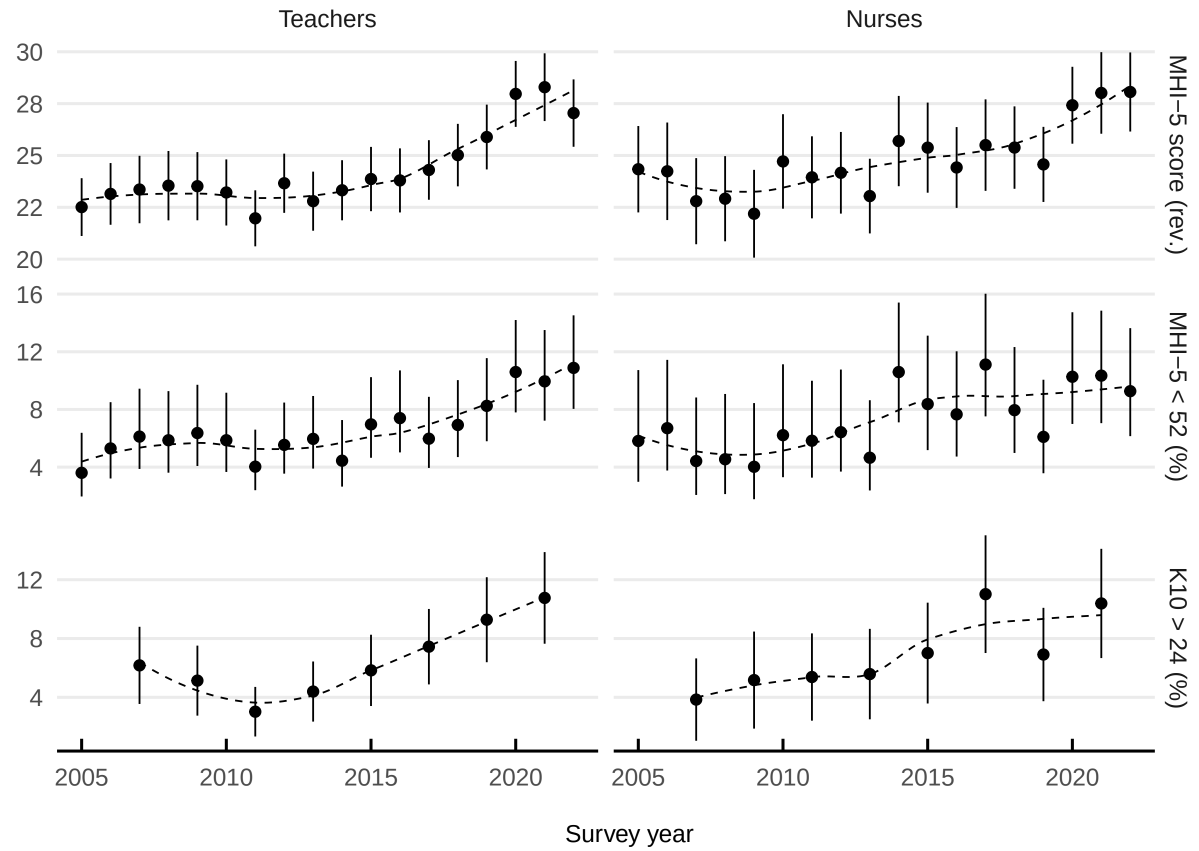

Figure A1 legend. Trends in mental ill-health by MHI-5 score (reversed), as well as prevalence of mood disorder by the prescribed cut-off scores for MHI-5 (annual) and K10 (biennial). Trends in each case were similar, indicating good correspondance between the continous mental health index used in the main analysis and clinical risk factors.

The correspondence between the continuous mental health score and the threshold mental-illness indicators (MHI-5 $<$ 52, K10 $>$ 24) demonstrates the full distribution of MHI-5 scores represents the prevalence of mental illness in this sample. In particular, these results support the inclusion of the continuous MHI-5 score in the main analysis.

The job demands component score, where higher scores represent the increasing severity of the psychosocial stressor, was constructed from the average of responses to the six items below. The six items were chosen from the items originally developed by Karasek [8,9], however more recent theoretical development has treated items related to learning new skills, using skills and taking initiative as job resources [10,11], so these were dropped and examined separately (see below).

Table A1. Job demands & complexity items

Code	Description	Response
jomms	My job is more stressful than I had ever imagined	Disagree [1] to Agree [7]
jompi	I fear that the amount of stress in my job will make me ill	Disagree [1] to Agree [7]
jomcd	My job is complex and difficult	Disagree [1] to Agree [7]
jomfast	I have to work fast in my job	Disagree [1] to Agree [7]
jomwi	I have to work very intensely in my job	Disagree [1] to Agree [7]
jomtime	I don't have enough time to do everything in my job	Disagree [1] to Agree [7]

The job control component score, where lower scores represent the absence of job control and so more exposure to the psychosocial stressor, was constructed from the average of responses to the eight items below.

Table A2. Job control items

Code	Description	Response
jomfd	I have freedom to decide how I do my own work	Disagree [1] to Agree [7]
jomls	I have a lot of say about what happens in my job	Disagree [1] to Agree [7]
jomfw	I have freedom to decide when I do my work	Disagree [1] to Agree [7]
jomdw	I have a lot of choice in deciding what I do at work	Disagree [1] to Agree [7]
jomflex	My working times can be flexible	Disagree [1] to Agree [7]
jombrk	I can decide when to take a break	Disagree [1] to Agree [7]
jomrpt	My job requires me to do the same things over and over again	Agree [1] to Disagree [7]
jomvar	My job provides me with a variety of interesting things to do	Disagree [1] to Agree [7]

Others have identified job insecurity as an important risk factor and linked with psychological distress [12,13]. Four items measured job security where higher scores indicated more security (jomwf was reverse scored). However this job component was dropped from the main analysis due to low prevalence among teachers.

Table A3. Job security items

Code	Description	Waves
jompf	I get paid fairly for the things I do in my job	Disagree [1] to Agree [7]
jomsf	I have a secure future in my job	Disagree [1] to Agree [7]
jomcsb	Company I work for will still be in business in 5 years	Disagree [1] to Agree [7]
jomwf	I worry about the future of my job	Agree [1] to Disagree [7]

Job resources can include job skills and the learning environment at work. Three items were included as putative indicators of job resources, where higher scores were assumed to indicate greater skill use. However mental health did not increase with greater skill use as expected, rendering the role of job skills ambiguous in the present report and so dropped from the main analysis.

Table A4. Job skill items

Code	Description	Waves
jomns	My job often required me to learn new skills	Disagree [1] to Agree [7]
jomus	I use my skills in current job	Disagree [1] to Agree [7]
jomini	My job requires me to take initiative	Disagree [1] to Agree [7]

Figure A2. Prevalence of job components

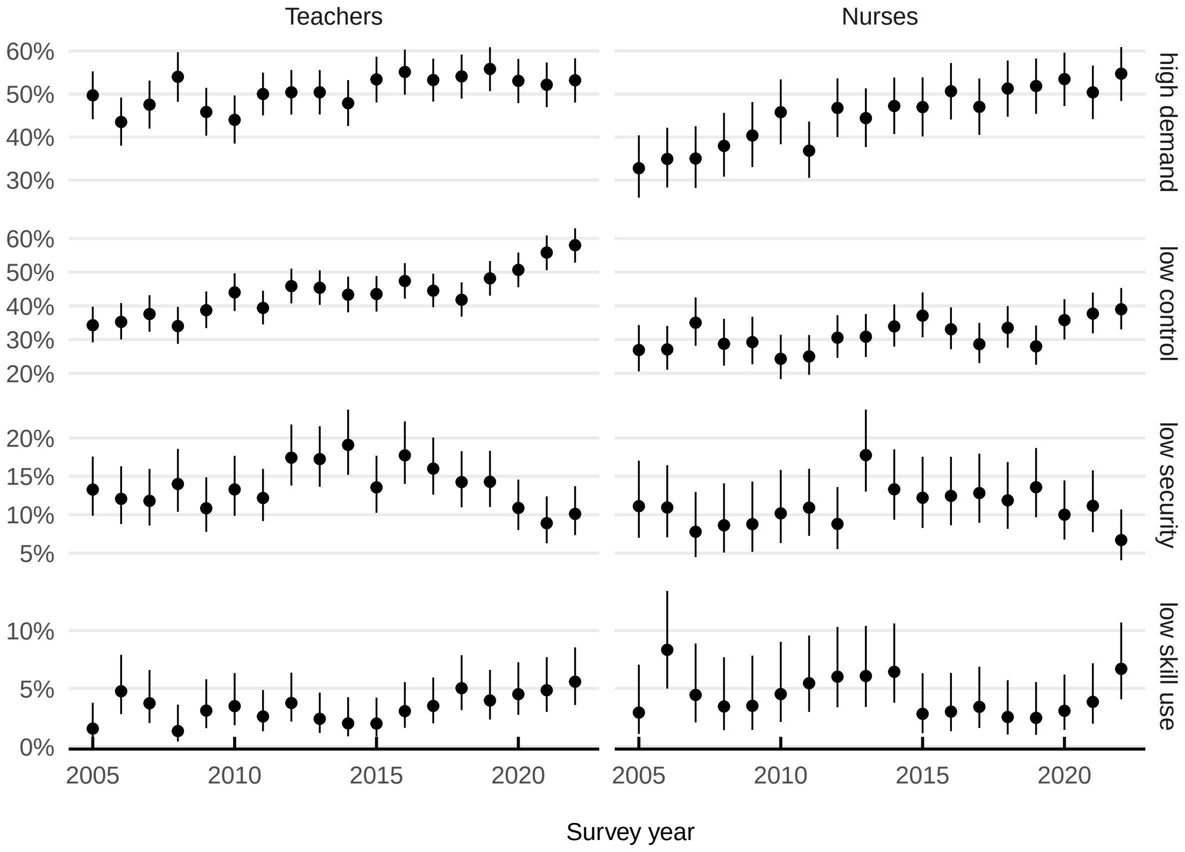

Figure A2 legend. Prevalence of high job demands and low job control among teachers was greater than 50 percent by the end of the period (2022). In comparison, the prevalence of low job security and low job skill resources was less than 15 percent.

Supplemental B. Model definitions and R-squared

Longitudinal models of annual change in MHI-5 scores (mental health) were constructed using the set of MHI-5 scores from each individual. MHI-5 scores were person-mean centered by subtracting the individual's mean MHI-5 score from each set to form the response variable (y_{it}), and so estimate the *within-subject effects* of time. The person-mean MHI-5 score was also included as a fixed effect (α_i), to address any time-invariant heterogeneity between people.

Models I to IV of MHI-5 scores included year as linear term as well as a penalised smooth additive term, where y_{it} represents the person-centered MHi-5 score for each person in each year, $f(\text{year}_t)$ is a smooth term to capture the average of the yearly changes in MHi-5 scores using cubic splines.

Model I:

$$y_{it} \sim \alpha_i + \beta_1 \text{year}_t + f(\text{year}_t) + \epsilon_{it}$$

Model II added terms for age, sex and job tenure, as well their linear interactions with time:

$$y_{it} \sim \alpha_i + \beta_1 \text{year}_t + f(\text{year}_t) + \beta_2 \text{sex}_i + \beta_3 \text{age}_{it} + \beta_4 \text{tenure}_{it} + \beta_4 \text{age}_{it} \cdot \text{year}_t + \beta_6 \text{tenure}_{it} \cdot \text{year}_t + \epsilon_{it}$$

Model III added linear and parametric terms for job control, where $f(\text{year}_t) \cdot \text{job component}_{it}$ represents the parametric interaction between the smooth effect of year and job component score:

$$y_{it} \sim \alpha_i + \beta_1 \text{year}_t + f_1(\text{year}_t) + f_2(\text{year}_t) \cdot \text{job control}_{it} + \beta_2 \text{sex}_i + \beta_3 \text{age}_{it} + \beta_4 \text{tenure}_{it} + \beta_4 \text{age}_{it} \cdot \text{year}_t + \beta_6 \text{tenure}_{it} \cdot \text{year}_t + \epsilon_{it}$$

Model IV included linear and parametric terms for job demand:

$$y_{it} \sim \alpha_i + \beta_1 \text{year}_t + f_1(\text{year}_t) + f_2(\text{year}_t) \cdot \text{job demand}_{it} + \beta_2 \text{sex}_i + \beta_3 \text{age}_{it} + \beta_4 \text{tenure}_{it} + \beta_4 \text{age}_{it} \cdot \text{year}_t + \beta_6 \text{tenure}_{it} \cdot \text{year}_t + \epsilon_{it}$$

Where y_{it} represents the person-centered MHi-5 score for each person in each year, $f(\text{year}_t)$ is a smooth term to capture the yearly changes in the average MHi-5 level using cubic splines, and $f(\text{year}_t) \cdot \text{job component}_{it}$ is another non-linear term representing smooth changes in the slope between the standardized job component scores and MHi-5 scores in each year over time.

Models were fit using mgcv (version 1.8-40)[14] in R (version 4.2.0)[15].

Figure B1. Proportion of variance explained in teacher mental health (R-squared) by job control (filled) and job demands (empty)

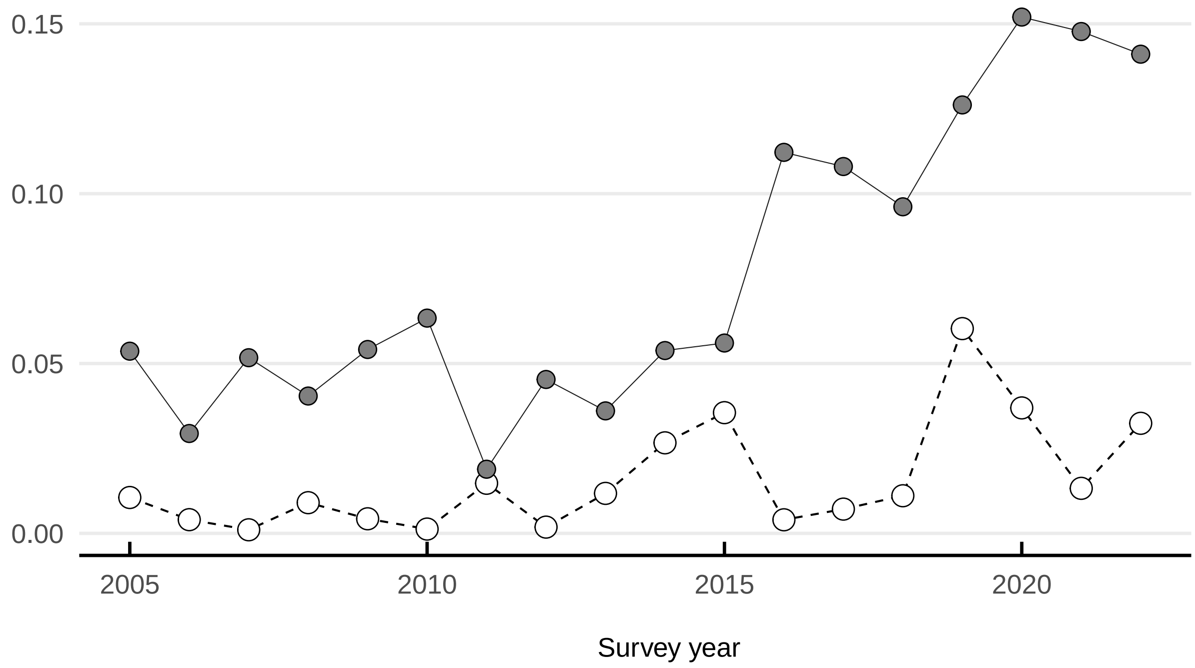

Figure B1 legend. The variance explained by OLS regression of job components on mental health each year. The variance explained by job demands (empty circles) increased over time faster than the variance explained by job control (filled circles).

Supplemental C: Alternate theories

Alternate explanations for the trend in teacher mental health include the Effort-Reward Imbalance model [16] and the Job demand-Resources model [10]. The Effort-Reward Imbalance model assumes the amount of subjective reward for work can act to reduce job stress or as an important buffer against job stress, while the Job Demand-Resources (JDR) model assumes contextual features of the job such as the learning environment and skill acquisition are a resource which can act as a buffer much like job control. Others have identified job insecurity as an important risk factor and linked with psychological distress [12,13]. Below we assumed teacher real wages and self-reported life satisfaction (i.e., subjective wellbeing or *happiness*) [17] were a relevant proxy for subjective reward to test whether the Effort-Reward Imbalance model explained the trend in teacher mental health. We also combined items related to learning new skills, using skills and taking initiative into a job skill score to test whether the Job Demand-Resource model explained the trend. Finally we tested the role of job security in the same manner. Sensitivity of teacher mental health to each of these factors was tested separately, in the same manner as job demand and job control in the main text. That is the partial-effect of time on mental health was estimated after holding each factor constant (job security, job skills, and happiness were held at the highest response level, while wage levels were held constant at the 75th percentile). The results are shown in the figure below.

Figure C1. Teacher mental health trend analysis partial effects

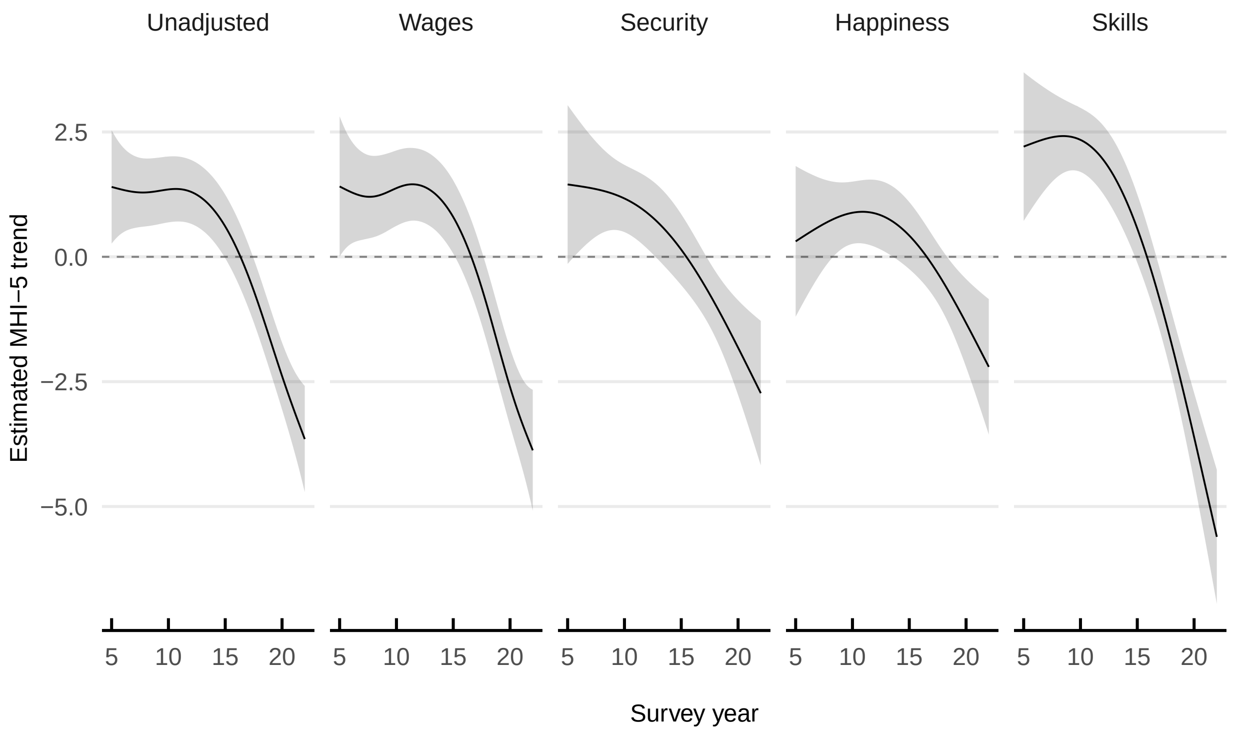

Figure C1 legend. Sensitivity of mental health over time to real wages, job security, happiness (subjective wellbeing) and job skill resources. Unadjusted is the total effect of time (survey year) on mental health, i.e., the teacher mental health trend.

There was little effect of real wages or job security on teacher mental health. Controlling for happiness (self-reported life satisfaction) ameliorated the decline in teacher mental health however the decline was still significant. Job skill resources increased the mental health decline, which was opposite to the JDR model

expectations. The absence of impact of subjective reward (either real wages or self-reported life satisfaction) on mental health did not support the Effort-Reward Imbalance model [16] as an alternative explanation for the decline in teacher mental health. Likewise, the impact of job skills did not improve mental health, which is *prima facie* inconsistent with the Job Demand-Resource (JDR) model [10,11]. However it may only indicate the items measured (Table A4) are not good proxies for job resources in this sample or setting, and so without further investigation we omit this evidence from the main report. Job security did not explain the trend in teacher mental health, which is not unexpected as it's prevalence was low among teachers (Figure A2).

Supplemental D: State-specific effects

In Australia, state and territory governments are responsible for running schools and setting local policy while the federal government provides the national strategy. For this reason, we considered whether including state effects in the trend models was warranted. State effects were included as separate penalized trends and the variation of each trend was estimated to determine whether it was significantly different from the overall trend. This is similar to deciding whether to include random effects in a mixed model by examining the variation around the random effect. The estimated smooth trends in mental health and the psychosocial job characteristics of teachers for each state and territory are shown below.

Figure D1. Estimated State-specific trends (±95%CI) in teacher mental health

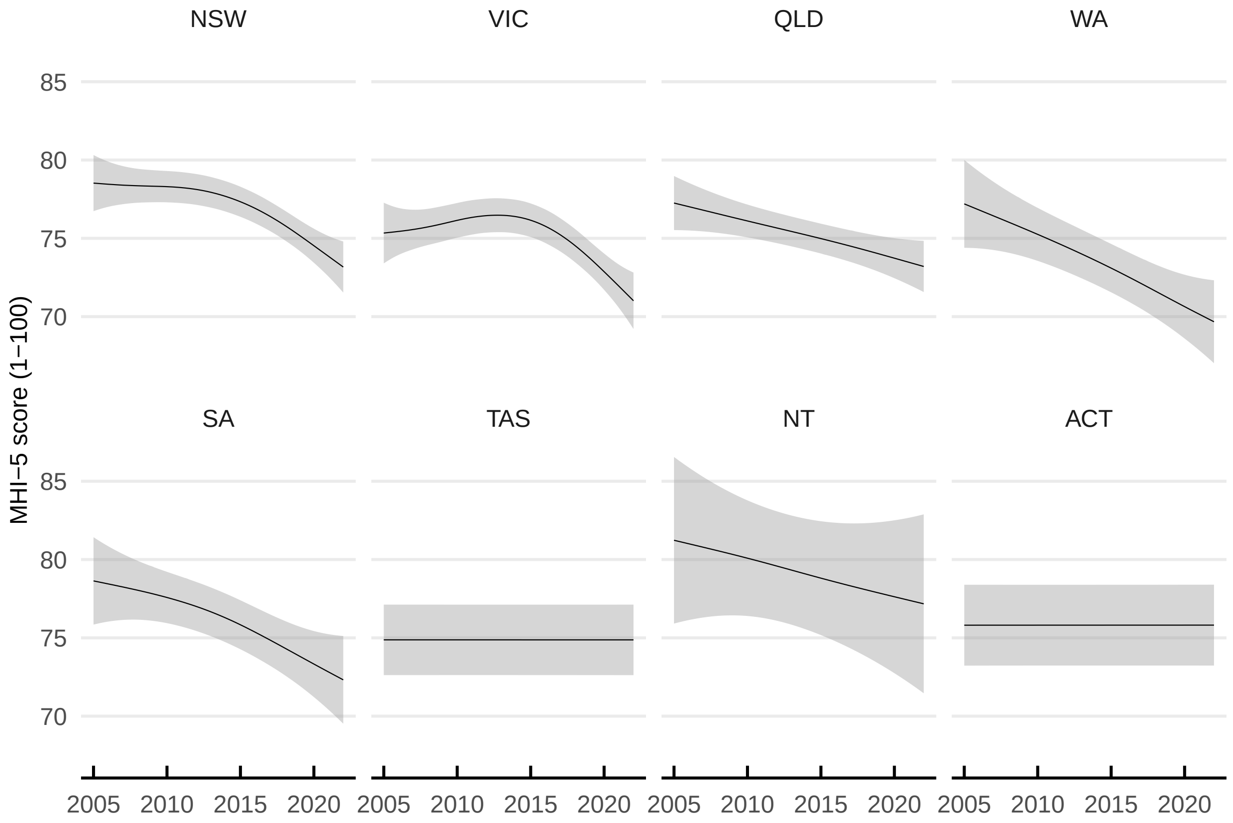

Figure D1 legend. Smooth trends in mental health for each state.

The state model estimated no positive mental health trends among the states, and a declining trend for six states, except for Tasmania (TAS) and the Australian Capital Territory (ACT), which represented less than 5 percent of our sample of teachers (n = 54/1,200). The table below shows the results of a likelihood ratio test against a null distribution of zero variance for each state, which reveals there was little evidence of any state or territory specific trends (smallest p-value = .061 for WA).

Table D1. Likelihood Ratio Test results for state-specific trends in teacher mental health ($H_0 = 0$ variance)

State/territory	edf	df	statistic	p.value
ACT	0.89	0.89	0.23	0.654
NSW	0.89	0.89	2.06	0.176
NT	3.08	3.80	1.45	0.247
QLD	1.67	2.11	1.39	0.220
SA	0.89	0.89	3.01	0.102
TAS	3.10	3.87	1.73	0.148
VIC	1.10	1.27	0.68	0.355
WA	0.89	0.89	3.94	0.061

*edf: estimated degrees of freedom

The results of a model comparison between a State-specific model and a non-specific model confirmed there was not sufficient evidence to warrant including State-specfic effects as an explanation [18]. The BIC difference between the two models was 98.2, which is a large difference favouring the non-specific model [19] and consistent with the non-specific model. The AIC difference between the two models was 1.9, which is a small difference and equivalent to a non-significant difference between models [19].

References

1 Chauvenet A, Buckley R, Hague L, *et al.* Panel sampling in health research. *The Lancet Psychiatry* 2020;**7**:840–1. doi:10.1016/S2215-0366(20)30358-8

2 Goodwin L, Ben-Zion I, Fear NT, *et al.* Are reports of psychological stress higher in occupational studies? A systematic review across occupational and population based studies. *PloS one* 2013;**8**:e78693. doi:10.1371/journal.pone.0078693

3 Kessler RC, Barker P, Colpe L, *et al.* Kessler psychological distress scale (K10). *Boston, MA: Harvard Medical School* 1996.

4 Cuijpers P, Smits N, Donker T, *et al.* Screening for mood and anxiety disorders with the five-item, the three-item, and the two-item mental health inventory. *Psychiatry research* 2009;**168**:250–5. doi:10.1016/j.psychres.2008.05.012

5 Hoeymans N, Garssen AA, Westert GP, *et al.* Measuring mental health of the dutch population: A comparison of the GHQ-12 and the MHI-5. *Health and quality of life outcomes* 2004;**2**:1–6. doi:10.1186/1477-7525-2-23

6 Rumpf H-J, Meyer C, Hapke U, *et al.* Screening for mental health: Validity of the MHI-5 using DSM-IV axis i psychiatric disorders as gold standard. *Psychiatry research* 2001;**105**:243–53. doi:10.1016/S0165-1781(01)00329-8

7 Batterham P, Sunderland M, Slade T, *et al.* Assessing distress in the community: Psychometric properties and crosswalk comparison of eight measures of psychological distress. *Psychological medicine* 2018;**48**:1316–24. doi:10.1017/S0033291717002835

8 Karasek Jr RA. Job demands, job decision latitude, and mental strain: Implications for job redesign. *Administrative science quarterly* 1979;285–308. doi:/10.2307/2392498

9 Karasek R, Brisson C, Kawakami N, *et al.* The job content questionnaire (JCQ): An instrument for internationally comparative assessments of psychosocial job characteristics. *Journal of occupational health psychology* 1998;**3**:322. doi:10.1037/1076-8998.3.4.322

10 Demerouti E, Bakker AB, Nachreiner F, *et al.* The job demands-resources model of burnout. *Journal of Applied psychology* 2001;**86**:499. doi:10.1037/0021-9010.86.3.499

11 Bakker AB, Demerouti E. The job demands-resources model: State of the art. *Journal of managerial psychology* 2007;**22**:309–28. doi:10.1108/02683940710733115

12 De Witte H, Näswall K. Objective vs subjective job insecurity: Consequences of temporary work for job satisfaction and organizational commitment in four European countries. *Economic and industrial democracy* 2003;**24**:149–88.

13 Richter A, Näswall K, Lindfors P, *et al.* Job insecurity and work–family conflict in teachers in s weden: Examining their relations with longitudinal cross-lagged modeling. *PsyCh Journal* 2015;**4**:98–111. doi:10.1002/pchj.88

14 Wood SN. Fast stable restricted maximum likelihood and marginal likelihood estimation of semi-parametric generalized linear models. *Journal of the Royal Statistical Society: Series B (Statistical Methodology)* 2011;**73**:3–36. doi:10.1111/j.1467-9868.2010.00749.x

15 R Core Team. *R: A language and environment for statistical computing.* Vienna, Austria: : R Foundation for Statistical Computing 2022. https://www.R-project.org/

16 Siegrist J. Adverse health effects of high-effort/low-reward conditions. *Journal of occupational health psychology* 1996;**1**:27. doi:10.1037/1076-8998.1.1.27

17 Diener E, Heintzelman SJ, Kushlev K, *et al.* Findings all psychologists should know from the new science on subjective well-being. *Canadian Psychology/psychologie canadienne* 2017;**58**:87. doi:10.1037/cap0000063

18 Shmueli G. To explain or to predict? *Statistical Science* 2010;**25**:289–310. doi:10.1214/10-STS330

19 Raftery AE. Bayesian model selection in social research. *Sociological methodology* 1995;111–63. doi:10.2307/271063
